# Direct Sensing of Endothelial Oxidants by Vascular Endothelial Growth Factor Receptor-2 and c-Src

**DOI:** 10.1371/journal.pone.0028454

**Published:** 2011-12-01

**Authors:** Monica Lee, Wing C. Choy, Md. Ruhul Abid

**Affiliations:** 1 Division of Cardiothoracic Surgery, Cardiovascular Research Center, Brown University Warren Alpert Medical School, Rhode Island Hospital, Providence, Rhode Island, United States of America; 2 Texas Tech University Health Sciences Center, School of Medicine, Lubbock, Texas, United States of America; 3 Department of Medicine, Beth Israel Deaconess Medical Center, Boston, Massachusetts, United States of America; Children's Hospital Boston & Harvard Medical School, United States of America

## Abstract

**Background:**

ADPH oxidase-derived reactive oxygen species (ROS) play important roles in redox homeostasis and signal transduction in endothelial cells (ECs). We previously demonstrated that c-Src plays a key role in VEGF-induced, ROS-dependent selective activation of PI3K-Akt but not PLCγ-1-ERK1/2 signaling pathways. The aim of the present study was to understand how VEGFR-2-c-Src signaling axis ‘senses’ NADPH oxidase-derived ROS levels and couples VEGF activation of c-Src to the redox state of ECs.

**Methodology/Principal Findings:**

Using biotinylated probe that detects oxidation of cysteine thiol (cys-OH) in intracellular proteins, we demonstrate that VEGF induced oxidative modification in c-Src and VEGFR-2, and that reduction in ROS levels using siRNA against p47^phox^ subunit of Rac1-dependent NADPH oxidase inhibited this phenomenon. Co-immunoprecipitation studies using human coronary artery ECs (HCAEC) showed that VEGF-induced ROS-dependent interaction between VEGFR-2 and c-Src correlated with their thiol oxidation status. Immunofluorescence studies using antibodies against internalized VEGFR-2 and c-Src demonstrated that VEGF-induced subcellular co-localization of these tyrosine kinases were also dependent on NADPH oxidsase-derived ROS.

**Conclusion/Significance:**

These results demonstrate that VEGF induces cysteine oxidation in VEGFR-2 and c-Src in an NADPH oxidase-derived ROS-dependent manner, suggesting that VEGFR-2 and c-Src can ‘sense’ redox levels in ECs. The data also suggest that thiol oxidation status of VEGFR-2 and c-Src correlates with their ability to physically interact with each other and c-Src activation. Taken together, these findings suggest that prior to activating downstream c-Src-PI3K-Akt signaling pathway, VEGFR-2-c-Src axis requires an NADPH oxidase-derived ROS threshold in ECs.

## INTRODUCTION

Reactive oxygen species (ROS) are oxidizing molecules that have unpaired electrons, such as superoxide (O_2_^⋅-^), hydroxyl anion (HO^⋅^), and nitric oxide (NO^⋅^), or that may not have free electrons but possess oxidizing ability, such as hydrogen peroxide (H_2_O_2_), hypochlorous acid (HOCl), and peroxynitrite (ONOO^-^). ROS are often considered as harmful metabolic by-products and have traditionally been implicated in the pathogenesis of cardiovascular diseases including hypertension, atherosclerosis, diabetic vasculopathy, and heart failure [1], [2], [3]. However, ROS, at physiological concentration, have recently been shown to be essential for signal transduction in vascular cells, including endothelial cells (ECs) [4], [5], [6], [7], [8], [9], [10], [11], [12], [13]. We and others have reported Rac1-dependent NADPH oxidase as a major source of superoxide in ECs and thus one of the important determinants of the redox content of the endothelium [14], [15], [16], [17]. Rac1-dependent NADPH oxidase consists of two membrane-bound components, gp91^phox^ (also known as Nox2) and p22^phox^, and several cytosolic regulatory subunits, including p47^phox^, p67^phox^, and the small GTPase Rac1. Upon agonist stimulation, NADPH oxidase transfers electrons from NAD(P)H to molecular oxygen to form O_2_^⋅-^. Recently, NADPH oxidase-derived ROS have been implicated in EC proliferation, migration, and angiogenesis [14], [15], [17].

Vascular endothelial growth factor (VEGF) is a potent EC-specific mitogen and chemotactic factor that is involved in wound repair, angiogenesis of ischemic tissue, tumor growth, microvascular permeability, vascular protection, and hemostasis [18], [19]. The VEGF family of proteins binds to three major receptor-type tyrosine kinases, Flt-1 (VEGF receptor-1), KDR/Flk-1 (VEGF receptor-2), and VEGFR-3 [20], [21]. VEGF activates a number of different intracellular signaling pathways, including phospholipase Cγ, protein kinase C, mitogen-activated protein kinase (MAPK)/extracellular signal–regulated kinase (ERK), non-receptor tyrosine kinase c-Src, and phosphatidyl inositol 3-kinase (PI3K)/Akt/protein kinase B in ECs.

In 2000, we reported that VEGF induced Rac1-dependent NADPH oxidase activity resulting in transient increase in ROS levels, and that NADPH oxidase-derived ROS are essential for proliferation and migration in ECs [15], [22]. These findings were supported by others in subsequent studies [16], [17]. More recently, we reported that reduction in NADPH oxidase activity resulted in inhibition of VEGF-induced activation of c-Src-PI3K-Akt-eNOS (but not PLCγ-1-ERK1/2) [23], reduction in NO synthesis and coronary arteriolar vasodilatation [24]. These suggested that an ROS threshold is required to selectively turn on c-Src-PI3K-Akt-eNOS by VEGF in ECs, where c-Src appears to play a major role in propagating signals from VEGFR-2 to downstream PI3K-Akt in a redox-dependent manner [23]. However, the precise mechanisms of signal transduction by which NADPH oxidase-derived ROS modulate some (e.g. c-Src) but not all (e.g. PLCγ-1-ERK1/2) post-VEGFR-2 signaling pathways are not known. In non-endothelial cells, transient inhibition of protein tyrosine phosphatases (PTPs) by oxidation of their catalytic cysteine thiols (SH-group) has been proposed to be the mechanism by which ROS help propagate receptor tyrosine kinase (RTK) signaling [25], [26], [27]. For example, ROS induced transient oxidation (sulfenic acid/cys-OH formation) of the cysteine residue(s) in the PTP, SHP-2, in response to PDGF that requires association with the PDGFR [25]. Recently, similar mechanisms have been proposed for VEGF signaling in ECs [28], [29], suggesting that ROS-mediated transient inhibition of the PTPs (e.g. PTP1B, SHP2, DEP-1) plays a permissive role in signal transduction by VEGFR-2. Our findings that NADPH oxidase-derived ROS are required for selective activation of some but not all signaling pathways downstream of VEGFR-2 [23] can not be explained by such generalized effect of PTP inhibition on VEGFR-2. Rather, our data suggested that VEGF-induced ROS were specifically required to channel post-VEGFR-2 signal to c-Src [23], whereas VEGFR-2-PLCγ-1-ERK1/2 signaling was independent of ROS. However, the molecular mechanisms by which ROS link VEGFR-2 with c-Src are not known. The goal of this study was to understand how VEGFR-2-c-Src signaling axis ‘senses’ ROS levels and couples VEGF activation of c-Src to the redox state of the endothelium. Here, we present novel findings that VEGF oxidizes cysteine thiol (cys-SH) in c-Src and VEGFR-2 in HCAEC. We also demonstrate that VEGF-induced oxidation of VEGFR-2, c-Src, and their intracellular interaction are specifically dependent on NADPH oxidase-derived ROS.

## RESULTS

### NADPH oxidase-derived ROS are required for signal propagation from VEGFR-2 to c-Src but not PLCγ-1

**Figure 1:**
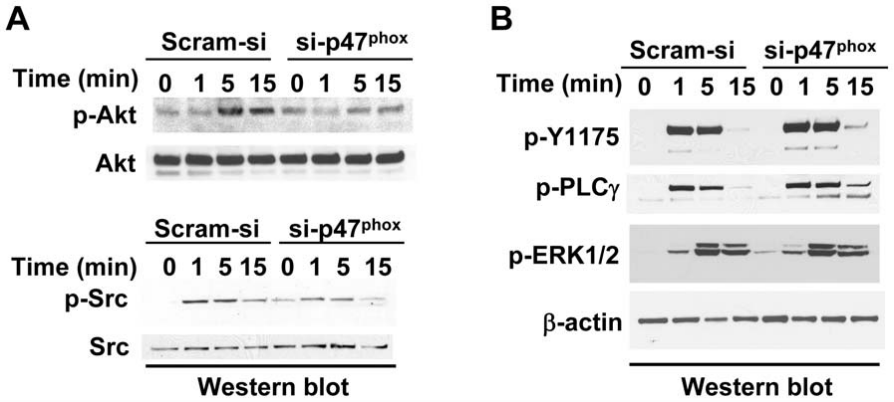
VEGF-induced activation of c-Src, Akt, but not VEGFR-2, PLCγ-1 and ERK1/2 requires NADPH oxidase-derived ROS. (A) Protein extracts from HCAEC transfected with control siRNA (Scram-si) or si-p47^phox^ were subject to Western blots as described in Materials and methods. Serum-starved HCAEC were treated with VEGF (50 ng/ml) for the times indicated. Membranes were sequentially blotted, stripped and re-probed with the phospho-specific antibodies as shown. Blots shown are representative of three independent experiments. (B) Same as in (A) except, the membranes were probed for phosphorylation of Y1175 VEGFR-2, Y783 PLCγ-1 and p42/44 ERK1/2. Anti-β-actin antibody was used as loading control.

We wanted to examine the role of NAPDH oxidase-derived ROS on VEGF-induced activation of VEGFR-2 and c-Src. To that end, total ROS levels were reduced by more than 50% in HCAEC by inhibiting NADPH oxidase using siRNA against p47^phox^ (si- p47^phox^) [23]. Reduction in intracellular ROS inhibited VEGF-mediated phosphorylation of Akt but not ERK1/2 in HCAEC (**Figure 1A** upper panel, and **Figure 1B**), suggesting that selective activation of some but not all VEGF signaling pathways requires ROS. In order to identify the redox-sensitive signaling intermediates that are upstream of Akt, we examined phosphorylation of VEGFR-2, PLCγ-1, and c-Src by VEGF in NADPH oxidase-knockdown HCAECs. We observed that whereas Y1175-VEGFR-2 (and total tyrosine, data not shown) and PLCγ-1 phosphorylation was not dependent on ROS (**Figure 1B**), c-Src phosphorylation was significantly inhibited in NADPH oxidase-knockdown HCAECs (**Figure 1A** lower panel). Generalized reduction in ROS levels in HCAEC using diphenylamine (DPI, 10 µM, for 30 mins) also resulted in selective inhibition of VEGF-mediated activation of c-Src and Akt but not PLCγ-1 or ERK1/2 (data not shown). Together, these findings suggest that ROS play a crucial role in selective propagation of signals from VEGFR-2 to downstream c-Src-PI3K-Akt but not PLCγ-1-ERK1/2.

### VEGF induces thiol oxidation of c-Src and VEGFR-2 in an ROS-dependent manner

**Figure 2:**
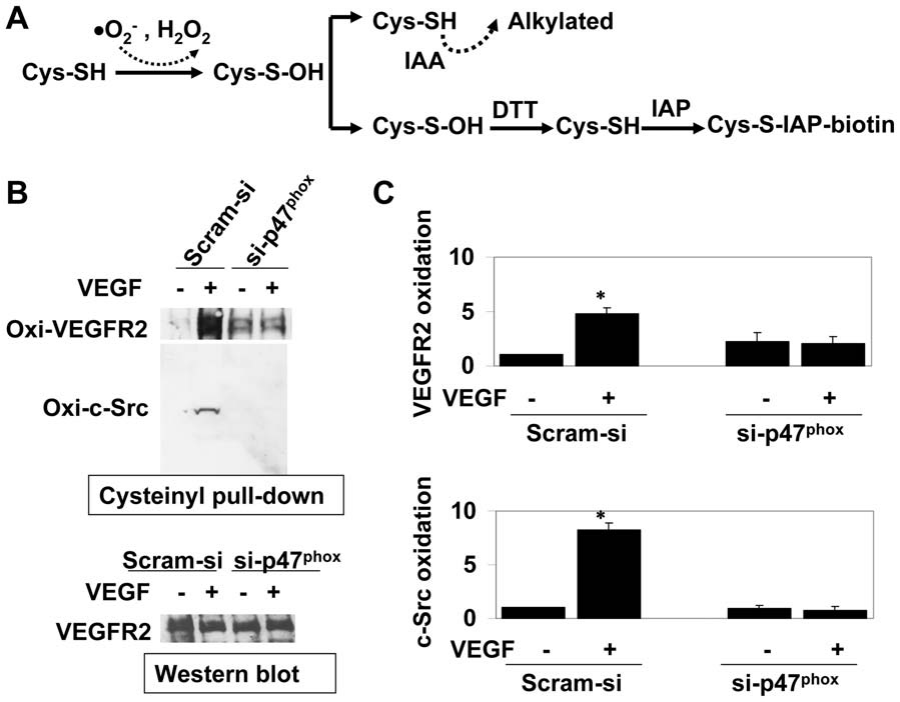
c-Src and VEGFR-2 are oxidized in VEGF-treated HCAEC in the presence of NADPH oxidase-derived ROS. (A) Schematic presentation depicting cysteinyl-labeling assay to determine oxidative modification in intracellular proteins. Non-oxidized protein thiols are alkyalated by IAA, oxidized thiols are reduced back to SH-moiety by DTT and subsequently biotinylated by IAP. Biotinylated proteins are then pulled-down by streptavidin-agarose followed by Western blots. (B) Upper panel: cysteinyl labeling assay to Identify thiol oxidation of proteins in VEGF-treated (50 ng/ml for 2 mins) HCAEC lysates using biotinylated IAP probe. HCAEC were transfected with Scram-si or si-p47^phox^ as indicated. After cell lysis in the presence of IAA followed by DTT treatment and IAP labeling, 1.5 mg biotinylated protein lysates were subject to immunoprecipitation using Streptavidin-agarose beads and immunoblotted using anti-VEGFR-2 and anti-c-Src antibodies. Lower panel: Western blot for VEGFR-2 using 50 µg of parallel HCAEC lysates as loading control. (B) Quantitative analyses of oxidized VEGFR-2 (upper panel) and c-Src (lower panel). Bar graph shows quantitative densitometric analysis of three independent cysteinyl labeling assays (as in A) using NIH J image (-fold change expressed in mean ± S.E.M.). *p<0.05 was considered statistically significant.

Since VEGF-induced activation of c-Src requires ROS in ECs [23] and thiol oxidation of c-Src has been shown to be associated with its kinase activity in non-endothelial cells [30], we examined whether VEGF treatment induces oxidation of cysteine residues in c-Src. To that end, we have utilized a modoified cysteinyl-labeling assay that is highly sensitive to sulfenic acid intermediates (cys-OH), which are formed initially during oxidative modification of cysteine SH-group (**Figure 2A**). This three-step assay includes alkylation of intracellular non-oxidized thiols, biotin-labeling of transiently oxidized cysteine (cys-OH) residues, and pull down of biotinylated proteins by streptavidin followed by Western blots. Two-minute incubation with VEGF induced c-Src oxidation (by 8.2± 0.68 fold) in HCAEC, whereas inhibition of NADPH oxidase-derived ROS using si-p47^phox^ completely blocked thiol oxidation of c-Src (**Figure 2B–C**). Similarly, ligand-induced thiol oxidation of VEGFR-2 (4.8± 0.74 fold) was also dependent on NADPH oxidase-derived ROS (**Figure 2B–C**). These findings suggest that VEGF induces oxidative modification in VEGFR-2 and c-Src, and that NADPH oxidase-derived ROS are essential for oxidation of these tyrosine kinases in HCAEC.

### VEGF-induced c-Src-VEGFR-2 interaction requires NADPH oxidase-derived ROS

**Figure 3:**
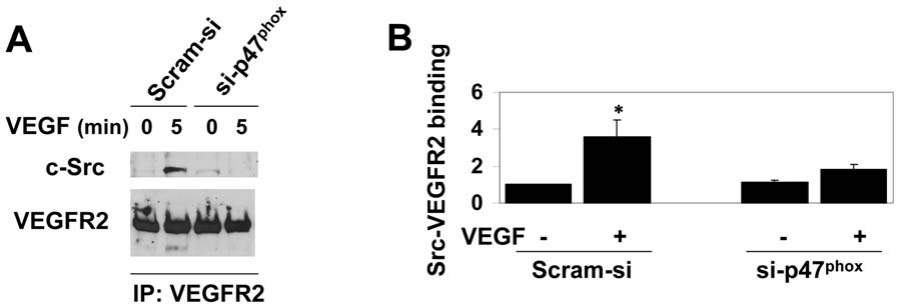
VEGF-induced interaction between VEGFR-2 and c-Src requires NADPH oxidase-derived ROS. (A) Co-immunoprecipitation (co-IP) assay using 1.2 mg protein lysates of HCAEC that were transfected with Scram-si or si-p47^phox^ and treated without or with VEGF (50 ng/ml for 5 min). IP was carried out using anti-VEGFR-2 antibody followed by immunoblotting using anti-c-Src (upper panel) and anti-VEGFR-2 (lower panel) antibodies. (B) Quantitative analyses of VEGFR-2-bound c-Src. Bar graphs show quantitative densitometric analysis of three independent experiments using NIH image J (-fold change expressed in mean ± S.E.M.). *p<0.05 was considered statistically significant.

**Figure 4:**
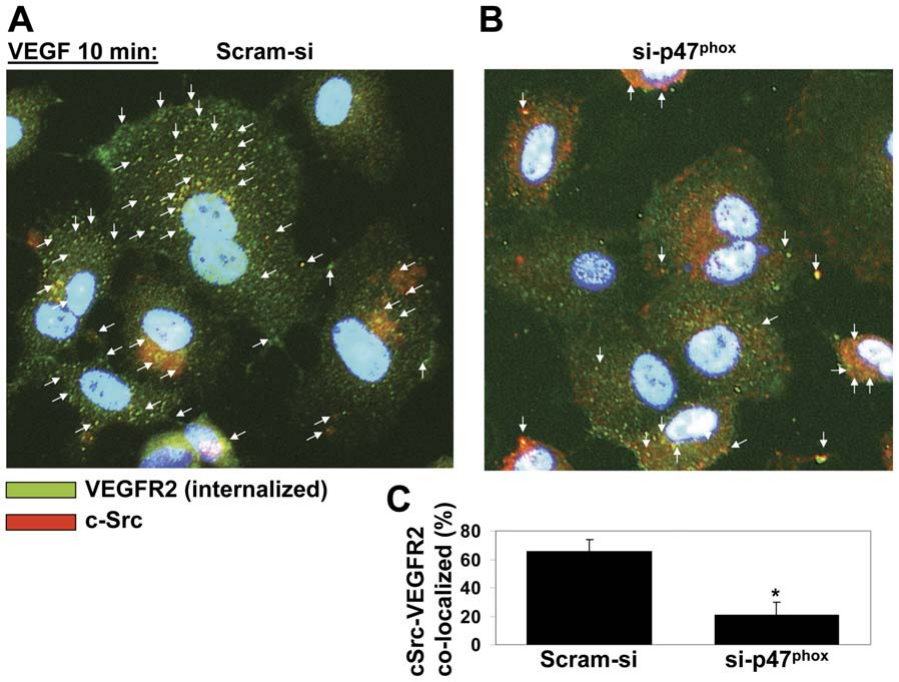
VEGF induces subcellular co-localization of c-Src and internalized VEGFR-2 in an ROS-dependent manner. HCAEC transfected with control (Scram-si) (A) or si-p47^phox^ (B) were immunofluorescently double labeled for internalized VEGFR-2 (green) and c-Src (red). VEGFR-2 on HCAEC was labeled with single chain E-tagged antibody (scFvA7, Fitzerald) as described in Materials and methods. After incubation with VEGF (50 ng/ml for 10 min), in order to remove the antibody from the cell surface, cells were placed on ice and acid washed. In permeabilized and fixed HCAEC, VEGFR-2 was detected with an AlexaFluor488-conjugated secondary antibody and is shown in green. c-Src was labeled with AlexaFluor647-conjugated secondary antibody (red) and nuclei with DAPI (blue). (B) Bar graphs show image analysis for colocalization events using the NIH Image J plugin (as described in Materials and methods). The graphs present the number of colocalization events normalized for the number of total VEGFR-2–positive immunofluorescence signals. Values are the mean of three experiments ± S.E.M., each containing numbers obtained from five random fields. *p<0.05 was considered statistically significant.

VEGFR-2 is known to bind c-Src upon ligand stimulation [31]. Since VEGF induces activation and thiol oxidation of both c-Src and VEGFR-2 (Figs. 1 and 2), we next wanted to examine whether interaction between c-Src and VEGFR-2 was dependent on NADPH oxidase-derived ROS. Co-immunoprecipitation assay showed that VEGF induced binding of c-Src to VEGFR-2 in HCAEC transfected with scrambled siRNA (Scram-si), whereas reduction in ROS significantly inhibited interaction between c-Src and VEGFR-2 in si-p47^phox^-transfected HCAEC (**Figure 3**). Immunofluorescence studies using antibody against internalized VEGFR-2 and c-Src demonstrated that reduction in ROS significantly reduced intracellular co-localization of these two tyrosine kinases (**Figure 4**).Together, these findings suggest that thiol oxidation of c-Src and VEGFR-2 may play an important role in VEGF-induced interaction between c-Src and VEGFR-2.

## DISCUSSION

The goal of the present study was to understand how VEGF signaling ‘senses’ ROS levels and tailors activation of a specific signaling pathway c-Src-PI3K-Akt, but not PLCγ-1-ERK1/2, to the redox state of the endothelium. Specifically, we have examined whether NADPH oxidase-derived ROS are required to induce oxidative modification in VEGFR-2 and c-Src by VEGF, and whether VEGF-induced interaction between VEGFR-2 and c-Src requires ROS. Using a biotinylated probe that binds proteins with cysteine sulfenic acid (cys-OH) modification, we show for the first time that VEGF induces thiol oxidation in VEGFR-2 and c-Src in HCAEC. Our data also demonstrate that ROS-induced, cysteine-thiol oxidation of the receptor (VEGFR-2) and the non-receptor (c-Src) tyrosine kinase correlate with their subcellular co-localization and physical interaction. These findings directly link endothelial redox status to the activation of a selective VEGF signaling pathway, namely c-Src-PI3K-Akt.

**Figure 5:**
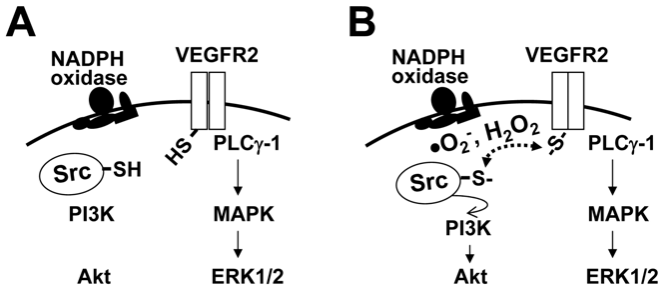
Proposed model: thiol oxidation may help propagate signal transduction from VEGFR-2 to downstream c-Src. (A) VEGF activation of VEGFR-2 and downstream PLCγ-1-ERK1/2 signaling pathway appears to be independent of ROS levels in ECs. (B) VEGF induces NADPH oxidase-derived ROS, which in turn oxidizes VEGFR-2 and c-Src. Thiol oxidation of these two tyrosine kinases appears to correlate with VEGF-induced activation of c-Src, and also with the sub-cellular colocalization and interaction between VEGFR-2 and c-Src. Dependence of VEGF-induced thiol oxidation and activation of c-Src on NADPH oxidase-derived ROS render downstream activation of PI3K-Akt redox-sensitive in HCAEC. In this model, VEGFR-2 and/or c-Src act as endothelial redox-sensors that determine whether downstream PI3K-Akt signaling pathway should be activated or not.

ROS act as second messengers by modulating protein functions through oxidation of cysteine thiols. The effect of thiol oxidation on the function of a protein is context-dependent. For example, ROS transiently inhibited PTPs by oxidizing catalytic cysteine thiol and thus allowed activation of signaling pathways downstream of PDGFR [25], [27]. On the contrary, ROS-induced thiol oxidation (and nitric oxide-mediated nitrosylation) at the C-terminus cysteine residues of c-Src was shown to regulate activity of the kinase in non-endothelial cells [32], [33], [34], [35]. However, a role for cysteine thiol oxidation in VEGF signaling pathways has not been reported. The current study shows a novel thiol oxidation mechanism by which NADPH oxidase-derived ROS may act to channel post-VEGFR-2 signaling specifically to downstream c-Src, and thus render c-Src-PI3K-Akt signaling ROS-dependent in ECs (Figure 5). However, our data do not identify specific cysteine residues that are oxidized by ROS in VEGFR-2 and c-Src. Although there are reports of redox-sensitive cysteine residues in the C-terminus of c-Src [32], [33], [34], [35], redox-sensitive cysteine residues in VEGFR-2 are not known. A sequence comparison study revealed two conserved cysteine residues in the cytoplasmic tail of VEGFR-2 among several species (Figure S1). Experiments are being carried out to determine whether one or more of these cysteine residues are oxidized by VEGF-induced ROS and whether intermolecular cysteine disulfide bridge formation plays a role in VEGFR-2-c-Src interaction in ECs. Disulfide bonds are usually formed by oxidation of cysteine's SH group as follows: 2 Cys-SH  =  Cys-S – S-Cys + 2 H^+^ + 2 e^-^. ROS including H_2_O_2_ oxidize cysteine residues in proteins to form this disulfide bond, where sulfenic acid (Cys-OH) formation acts as an intermediate product of oxidation [36].

It is interesting to note that although activation of PLCγ-1 is not ROS-dependent (Figure 1), VEGF induced thiol oxidation in PLCγ-1 in an NADPH oxidase-derived ROS-independent manner (Figure S2). Together, these data suggest that other sources of ROS may be involved in this phenomenon, thus rendering VEGF-induced activation of PLCγ-1-ERK1/2 signaling independent of NADPH oxidase-derived ROS. Alternate plausible explanation is that oxidation may not play significant role in VEGF-induced activation of PLCγ-1.

Upon activation, internalization and endocytic trafficking of VEGFR-2 are critical for proper signal propagation in ECs [28], [37], [38]. Our preliminary data demonstrated that, although internalization of VEGFR-2 was not dependent on ROS, subcellular trafficking through early stage endosomes (EEA-1 positive) was affected by NADPH oxidase inhibition (Figure S3). However, this finding does not address the question whether ROS play any role in the internalized receptor's recycling to the membrane (through endocytic pathway) and/or degradation. It is plausible that redox-dependent endocytic trafficking of the receptor is a pre-requisite for proper co-localization and interaction between VEGFR-2 and c-Src in ECs. Further experiments are required to address this question.

In summary, the data presented in this study demonstrate for the first time that VEGF induces thiol oxidation in VEGFR-2 and c-Src in an NADPH oxidase-derived ROS-dependent manner (Figure 5). Subcellular localization and interaction between these two tyrosine kinases are associated with their oxidation status. Although interaction with c-Src was inhibited (Figure 3), phosphorylation and internalization (Figure 1, Figure S2) of VEGFR-2 were not affected by reduction in ROS levels, suggesting that thiol oxidation of the receptor may play a specific role in c-Src activation. Further studies are required to elucidate the mechanisms by which thiol oxidation mediates interaction between these two tyrosine kinases and activation of downstream c-Src. Experiments are now being carried out to examine the involvement of the conserved cysteine residues in the cytoplasmic domain of VEGFR-2 in this process. In order to determine functional significance of these findings, in vivo studies are also undergoing to detect in situ thiol oxidation in VEGFR-2 and c-Src in intact vascular endothelium.

## MATERIALS AND METHODS

### Cell culture and reagents

Human coronary artery endothelial cells (HCAEC) were obtained from Clonetics and grown in Endothelial Growth Medium-2-MV (EGM-2-MV) BulletKit (Clonetics, San Diego, CA) at 37°C and 5% CO_2_. Endothelial cells from passage 3 to 6 were used for all experiments. Cells were serum starved in 0.5% fetal bovine serum (FBS) for 16 h prior to treatment with 50 ng/mL human VEGF-A_165_ (PeproTech Inc, Rocky Hill, NJ). For the detection of internalized VEGFR-2 by immunofluorescence, anti–human VEGFR-2 (single chain recombinant; clone scFvA7 with E tag; RDI and Fitzgerald) was used. Rabbit polyclonal C-1158 (sc504; Santa Cruz Biotechnology, Inc.) was used for Western blotting detection of VEGFR-2. Antibodies to tyrosine-phosphorylated VEGFR-2, phospho-Y1175, was from Cell Signaling. Antibody to EEA-1 was goat polyclonal N-19 (sc-6415) from Santa Cruz Biotechnology, Inc. Antibodies against phospho-S473-Akt, total Akt, phospho-Y783-PLCγ-1, phospho-p42/44 MAPK, phospho-Y416-c-Src, and total c-Src were from Cell Signaling. Zeba desalt spin columns (89889), IAA (35603), and IAP were from Thermo Scientific (Pierce Biotechnology).

### siRNA transfection

HCAEC were grown to 70–80% confluence in 6-cm plates and transfected with 100 nM ON-TARGETplus si-RNA against p47^phox^ (Dharmacon, Lafayette, CO) or scrambled siRNA (Scram-si) in Opti-MEM containing 10 µg/mL Lipofectin (Invitrogen, Carlsbad, CA) for 5 h. The cells were then incubated in EGM-2-MV medium for 24 h following serum starvation in endothelial basal medium (EBM-2; Clonetics) containing 0.2% serum for 16 h prior to VEGF treatment.

### Western blotting

HCAEC were harvested for total protein and Western blots were carried out as previously described [39]. Phospho-specific antibodies against Y416-c-Src, Ser-473 Akt, PLCγ-1, and ERK1/2 were purchased from Cell Signaling (Beverly, MA). Anti-β-actin antibodies were from Sigma. Western blots were performed using cell extracts prepared from three independent experiments.

### Cysteinyl labeling/thiol oxidation assay

Cysteinyl labeling assay, a three-step method in which reversibly oxidized cysteine (Cys) residues are specifically biotinylated and enriched to quantitatively measure thiol oxidation [27]. The steps are: (i) the reduced/non-oxidized Cys residues are irreversibly alkylated and excluded from the reaction by iodoacetic acid (IAA); (ii) IAA-cleared lysates are then incubated with 1 mM DTT to reduce the oxidized Cys residues (cyclic sulfenamide and sulfenic acid forms of Cys), which were protected from alkylation in the previous step, back to their thiolate states; and (iii) these thiol residues (that were initially oxidized inside the cells and protected from alkylation by IAA, but now are in the reduced state) are then biotinylated with sulfhydryl-reactive iodoacetylpolyethylene oxide (IAP, Thermo Scientific) probe (5 mM). The biotinylated proteins are then enriched by using streptavidin-agarose beads and analyzed by Western blots using antibodies against proteins of interest. The signal intensity of the biotinylated bands directly correspond to the oxidation status of the cysteine residues of the proteins. VEGFR-2 and c-Src were subsequently identified by immunoblotting with anti-VEGFR-2 and anti-c-Src antibodies (Cell Signaling), respectively. The cell lysis buffer used contained the following: 25 mM sodium acetate (pH 5.5), 1% Nonidet P-40, 150 mM NaCl, 10% (vol/vol) glycerol, 25 µg/ml aprotinin, 25 µg/ml leupeptin, supplemented with freshly prepared 10 mM IAA, 100 µg/ml catalase, and superoxide dismutase. After 1 h at room temperature to allow complete alkylation of free thiols, 1 mg of cell lysate was loaded onto desalting columns that had been equilibrated with IAA-free lysis buffer. Columns were centrifuged at 2,000 × g for 2 min at 4°C to remove IAA. The flow-through of the desalting columns (IAA-cleared lysates) was then incubated with 1 mM DTT for 30 min on a shaker at room temperature. 5 mM IAP (biotinylated probe) were added to bind reduced thiols, and biotinylated proteins were pulled down using streptavidin–agarose beads overnight at 4°C. The precipitated beads were washed with PBS and resuspended in 20 µl of 4× Laemmli sample buffer, heated at 90°C for 3 min before loading onto SDS gel.

### Colocalization of VEGFR-2 with c-Src or EEA1-positive endosome by immunofluorescence

HCAEC were plated on fibronectin coated glass-bottom chamber slides (Lab-Tek II, Thermo Scientific) and starved overnight in 0.2% FBS containing EBM-2. Cells were pre-cooled for 30 min on ice and treated with 10 µg/ml single chain recombinant scFvA7 with E tag antibody for 1 h on ice with gentle agitation [40], [41]. This single chain antibody to the extracellular domain of human VEGFR-2 is devoid of biological activity and does not affect basal or VEGF-stimulated phosphorylation and internalization of VEGFR-2. Before stimulation, cells were washed with ice-cold 0.2% FBS in EBM-2 medium to remove unbound antibody, and fresh serum-starvation (0.2% FBS in EBM-2) medium was added. HCAEC were stimulated with 50 ng/ml VEGF and transferred to 37°C. After incubation with VEGF, in order to remove the antibody from the cell surface, cells were placed on ice and acid washed (three washes with ice-cold 50 mM glycine in Ca^2+^/Mg^2+^ HBSS, pH 2.5, and two washes with Ca^2+^/Mg^2+^ HBSS, pH 7.5). Fixation and permeabilization were carried out in 1% PFA and 0.02% saponin, respectively. Cells were then labeled with goat anti-EEA1 antibody or anti-c-Src antibody overnight, followed by chick anti-goat alexa647 (red) secondary antibody. For the recombinant E-tagged anti–human VEGFR-2, rabbit anti–E-tag (Abcam) followed by AlexaFluor488-conjugated (green) donkey anti–rabbit (Invitrogen) were used. Samples were observed under a fluorescence Nikon Eclipse E800 microscope and a Spot digital camera. Co-localization was quantified using the Image J colocalization plugin. Co-localization events were normalized for the number of VEGFR-2-positive compartments. For both channels, the best-fit lower threshold value was used to remove background signal using threshold tool and counting one-pixel dimension particles. Means were taken from five random fields from each of 3 experiments.

### Statistical analysis

All values are presented as mean ± SEM where appropriate. Statistical significance between two groups was determined by use of a paired *t*-test, and values of p<0.05 were considered significant.

## Supporting Information

Figure S1 **VEGFR-2 from different species demonstrates conservation of cys 1201 and cys 1208 in the cytoplasmic tail.**
(TIFF)

Figure S2 **VEGF-induced thiol oxidation in PLCγ-1 does not require NADPH oxidase-derived ROS.** Cysteinyl labeling assay to Identify thiol oxidation of PLCγ-1 in VEGF-treated (50 ng/ml for 2 mins) HCAEC lysates using biotinylated IAP probe. HCAEC were transfected with Scram-si or si-p47^phox^ as indicated. After cell lysis in the presence of IAA followed by DTT treatment and IAP labeling, 1.5 mg biotinylated protein lysates were subject to immunoprecipitation using Streptavidin-agarose beads and immunoblotted using anti-PLCγ-1 antibody.
(TIF)

Figure S3 **Colocalization of internalized VEGFR-2 with EEA-1-positive early endosome is redox-sensitive.** HCAEC transfected with control (Scram-si) (A) or si-p47^phox^ (B) were double labeled for internalized VEGFR-2 (green) and EEA-1 (red). Internalized VEGFR-2 was labeled for immunofluorescence assay as described in the Legend of Figure 4A and is shown here in green. EEA-1 positive endosomes were labeled with AlexaFluor647-conjugated secondary antibody and is shown in red. Nuclei were stained with DAPI (blue). (C) Bar graphs show image analysis for colocalization events using the NIH Image J plugin as described in the Legend of Figure 4B. The graphs present the number of colocalization events normalized for the number of VEGFR-2–positive compartments. Values are the mean of three experiments ± S.E.M., each containing numbers obtained from five random fields. *p<0.05 was considered statistically significant.
(TIF)
