# Supplementary figures and images for: Direct Sensing of Endothelial Oxidants by Vascular Endothelial Growth Factor Receptor-2 and c-Src
Source: PLoS One. 2011 Dec 1;6(12):e28454. doi: 10.1371/journal.pone.0028454 (PMC3228784; doi:10.1371/journal.pone.0028454)

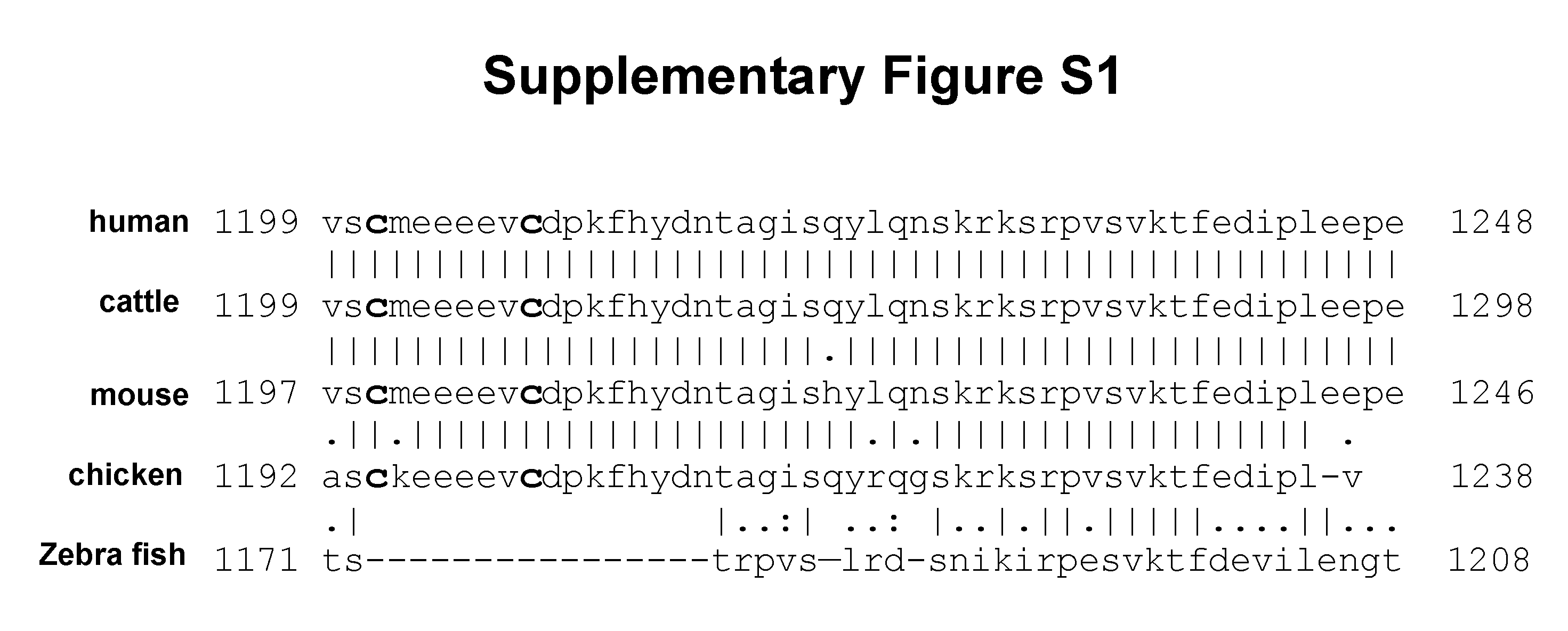

Supplement: Figure S1 — VEGFR-2 from different species demonstrates conservation of cys 1201 and cys 1208 in the cytoplasmic tail. (TIFF) [file pone.0028454.s001.tiff]

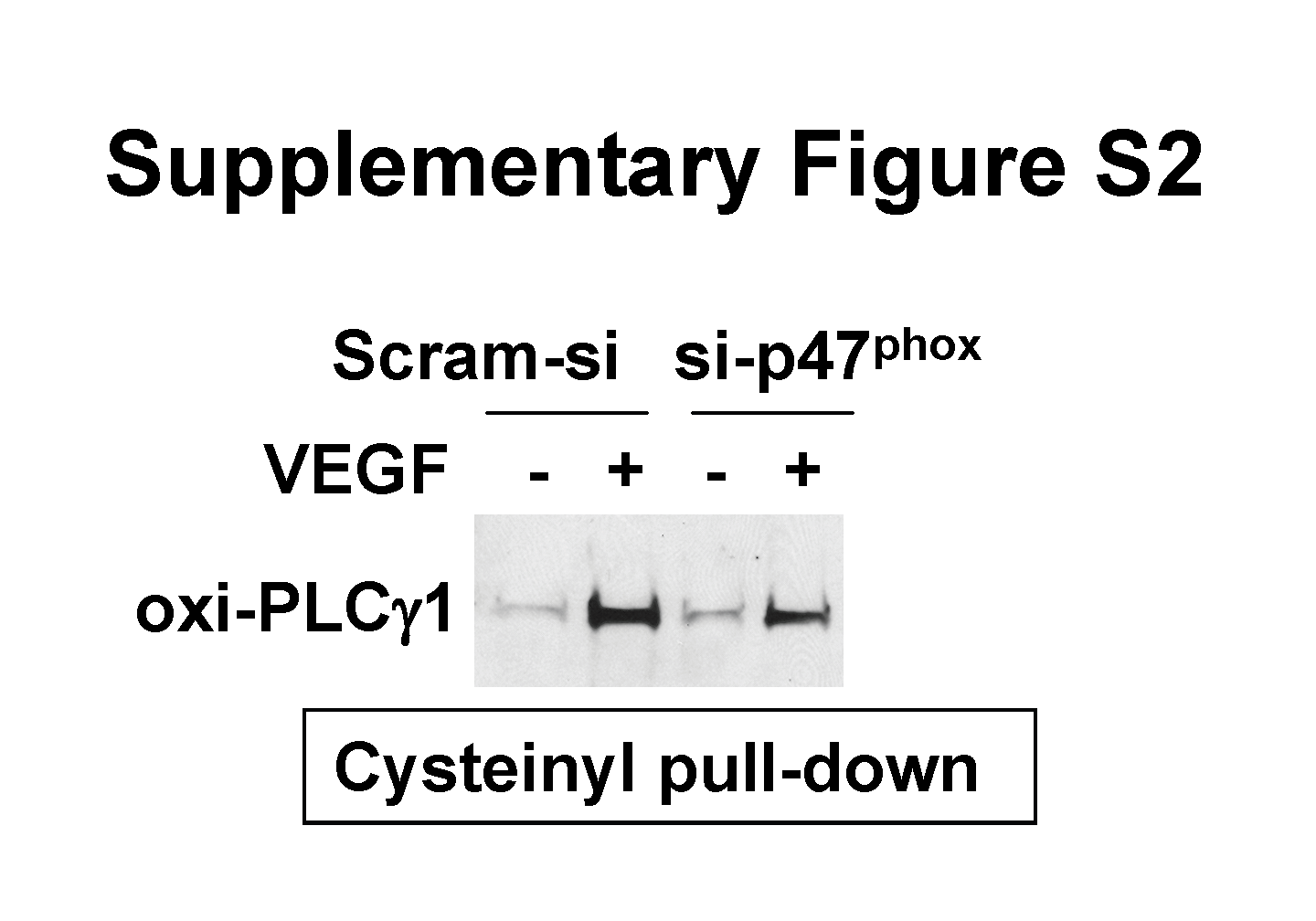

Supplement: Figure S2 — VEGF-induced thiol oxidation in PLCγ-1 does not require NADPH oxidase-derived ROS. Cysteinyl labeling assay to Identify thiol oxidation of PLCγ-1 in VEGF-treated (50 ng/ml for 2 mins) HCAEC lysates using biotinylated IAP probe. HCAEC were transfected with Scram-si or si-p47phox as indicated. After cell lysis in the presence of IAA followed by DTT treatment and IAP labeling, 1.5 mg biotinylated protein lysates were subject to immunoprecipitation using Streptavidin-agarose beads and immunoblotted using anti-PLCγ-1 antibody. (TIF) [file pone.0028454.s002.tif]

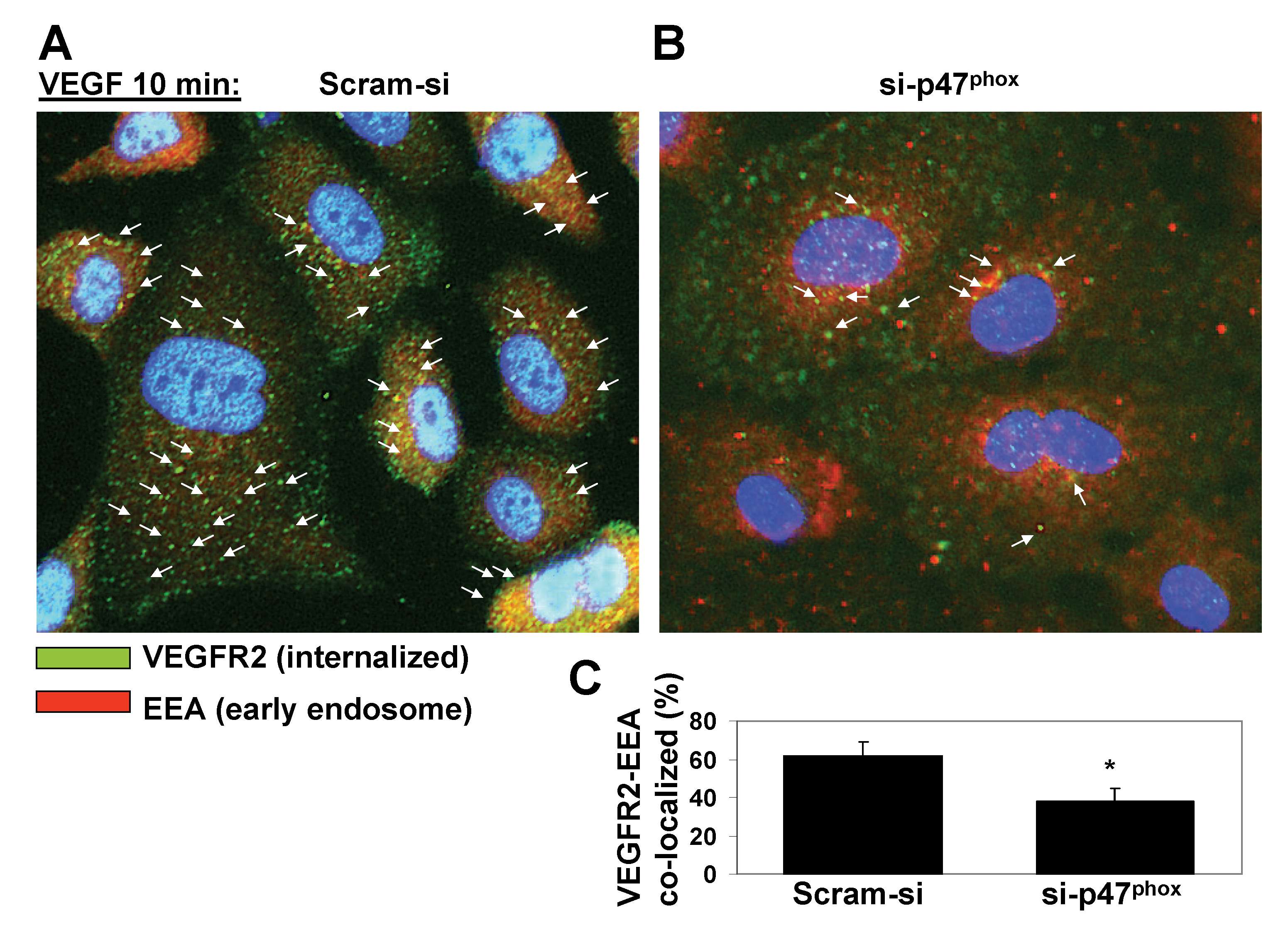

Supplement: Figure S3 — Colocalization of internalized VEGFR-2 with EEA-1-positive early endosome is redox-sensitive. HCAEC transfected with control (Scram-si) (A) or si-p47phox (B) were double labeled for internalized VEGFR-2 (green) and EEA-1 (red). Internalized VEGFR-2 was labeled for immunofluorescence assay as described in the Legend of Figure 4A and is shown here in green. EEA-1 positive endosomes were labeled with AlexaFluor647-conjugated secondary antibody and is shown in red. Nuclei were stained with DAPI (blue). (C) Bar graphs show image analysis for colocalization events using the NIH Image J plugin as described in the Legend of Figure 4B. The graphs present the number of colocalization events normalized for the number of VEGFR-2–positive compartments. Values are the mean of three experiments ± S.E.M., each containing numbers obtained from five random fields. *p<0.05 was considered statistically significant. (TIF) [file pone.0028454.s003.tif]
